# Simultaneous flow dynamics in small and great thoracic vessels during physiological stress tests and normal breathing using real-time cardiac magnetic-resonance

**DOI:** 10.1186/1532-429X-16-S1-P9

**Published:** 2014-01-16

**Authors:** Jan M Sohns, Martin Fasshauer, Johannes T Kowallick, Andreas Schuster, Wieland Staab, Arun Joseph, Shoun Zhang, Dirk Voit, Sebastian Schaetz, Klaus-Dietmar Merboldt, Michael Steinmetz, Christina Unterberg-Buchwald, Jens Frahm, Joachim Lotz

**Affiliations:** 1 Insitute for Diagnostic and Interventional Radiology, UMG Goettingen, Goettingen, Lower-Saxony, Germany; 2 Max-Planck-Institut für biophysikalische Chemie, NMR Forschungs GmbH, Goettingen, Lower-Saxony, Germany; 3 UMG Goettingen, Cardiology and Pneumology, Goettingen, Lower-Saxony, Germany; 4 German Centre for Cardiovascular Research, DZHK, Goettingen, Lower-Saxony, Germany; 5 UMG Goettingen, Pediatric Cardiology and Intensive Care Medicine, Goettingen, Lower-Saxony, Germany

## Background

Aim of this study was to measure blood flow dynamics in the descending aorta (AD) and azygos vein (AzV) using Muller (MM) and Valsalva (VM) maneuver as physiological stress tests and normal breathing in real-time cardiac magnetic resonance imaging (RT-CMR).

## Methods

Blood flow was measured in n = 20 healthy volunteers in the AD and AzV with RT-CMR including an image acquisition of under 40 ms and undersampled further development of established FLASH sequences. The volunteers were instructed to perform MM as well as VM in a 3T MR system, TR/TE were 3.44/2.76 ms. Pressure changes were digitally monitored to ensure sufficient pressure gradients.

## Results

Muller maneuver: At the beginning of MM, flow per heart beat (hb) in the AD (56 ± 9.4 SD ml/hb ~ 100%) decreased significantly (p. < 001, to 46 ± 10.4 ml/hb ~ 82% ± 13 ml/hb at early strain phase). In the AzV flow per hb increased significantly (p < .001) during MM (normal breathing 1.8 ± 1.5 ml/hb ~ 100% vs. early strain 5.5 ± 3.6 ml/hb ~ 309% ± 555 ml/hb). Heart rate increased significantly (p. < 001, normal breathing 74 ± 9.9 bpm ~ 100% vs. early strain 79 ± 12.6 bpm ~ 108% ± 8 bpm). Average peak velocity decreased significantly (p < .001) in AD (normal breathing 62 ± 11.8 cm/s ~100% vs. early strain 52 ± 11.6 cm/s ~ 84% ± 10 cm/s) and increased significantly (p < .01) in the AzV (normal breathing 16 ± 4.4 cm/s ~ 100% vs. early strain 25 ± 8.4 cm/s ~ 162% ± 56 cm/s). Average area in AD (p < .001) and AzV (p < .05) increased during MM. Valsalva maneuver: During VM flow per hb in the AD (regular breathing 56 ± 9.8 ml/hb ~ 100%) decreased significantly (p. < 001) until the end of VM (late strain 23 ± 10.9 ml/hb ~ 36% ± 18). Similar significant changes could be found during VM for average peak velocity (regular breathing 62 ± 12.9 cm/s ~ 100% vs. 42 ± 11.8 cm/s late strain ~ 61% ± 25). Heart rate increased significantly compensatory (p. < 001) from 75 ± 9.1 bpm during regular breathing (~100%) to 99 ± 17 bpm at late strain phase (~ 119% ± 43). Flow per hb changed significantly (p < .001) in the AzV from 2.2 ± 1.4 ml/hb (~ 100%) to 2.0 ± 2.1 ml/hb (~ 111% ± 223). Average peak flow velocity changed less significant (p < .05) from 17 ± 3.4 cm/s (~ 100%) to 14 ± 5.7 cm/s (~ 75% ± 43). The luminal area of the AzV changed non-significantly, in AD significantly (p < .001). All values are with standard deviation, ± SD.

## Conclusions

RT-CMR reliably and simultaneously determines flow dynamics during physiological stress. Compared to routinely used Cine-CMR it is able to show beat to beat variability in great and small thoracic vessels in detail. In comparison to echocardiography, it enables the investigator to measure flow simultaneously in thoracic descending aorta and azygos vein. CMR might provide new diagnostics insights into aortic diseases or portocaval hypertension in future.

## Funding

This work was funded by the Deutsche Forschungsgemeinschaft, DFG (LO 1773/1-1).

**Table 1:** Patient's characteristics

Type	Total ± SD
Age	33.86 ± 12.75

Weight	71.45 ± 13.69

Height	1.76 ± 0.11

Body mass index	22.82 ± 2.62

Cardiovascular risk factors	n = 1*

Gender	male (12/8)

* One proband with nicotin abusus (many pack years)

**Figure 1:**
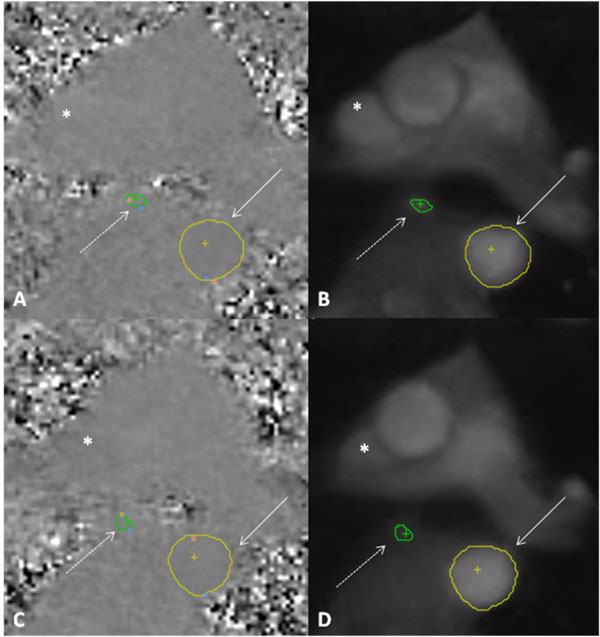
**Flow in the aorta descendens (arrow, yellow contour) and vena azygos (dashed arrow, green contour), images in systole (28-years old healthy male volunteers)**. Normal breathing (A-B) and Valsalva maneuver (C-D). Lumen of the aorta und vena azygos is compressed during Valsava (C, D), especially reduced in the superior vena cava (white stars). The luminal area and diameter of thoracic vessels is reduced under elevated intra-thoracic pressure. Scan area (localizers) were adapted to Valsalva and normal breathing, because mediastinal and heart structures are moved during variation of the intra-thoracic pressure.

